# Chemometric-assisted UV spectrophotometric methods for determination of miconazole nitrate and lidocaine hydrochloride along with potential impurity and dosage from preservatives

**DOI:** 10.1186/s13065-025-01447-9

**Published:** 2025-03-28

**Authors:** Esraa S. Ashour, Ghada M. El-Sayed, Maha A. Hegazy, Nermine S. Ghoniem

**Affiliations:** Analytical Chemistry Department, Faculty of Pharmacy, Cairo University, Kasr-El-Aini, Cairo, 11562 Egypt

**Keywords:** Miconazole, Lidocaine, Toxic impurity, Principal component regression, Partial least squares, Backward interval partial least squares

## Abstract

Three accurate, simple, and precise chemometric techniques, principal component regression (PCR), partial least squares (PLS), and backward interval partial least squares (biPLS) were used to resolve the severely overlapped UV spectra of miconazole nitrate (MIC) and Lidocaine hydrochloride (LDC) along with the toxic impurity of LDC; dimethyl aniline (DMA) and the two inactive ingredients; methyl paraben (MTP) and saccharin sodium (SAC). The concentration ranges of the developed models were found to be (2.40–12.00 µg/mL) for LDC and MIC, (1.50–7.50 µg/mL) for DMA and MTP, and (2.00–6.00 µg/mL) for SAC. The proposed methods were found to be green, rapid, and were effectively used to analyze the studied compounds in both laboratory-prepared mixtures and antifungal oral gel, where no impurity was detected. The obtained results revealed that PLS algorithm was superior to PCR depending on the lowest root mean square error of prediction (RMSEP) and correlation coefficient values (r). The biPLS model, constructed with [3, 4, 5, 6, 8, and 9] subintervals, is considered the most efficient model with the lowest number of latent variables. biPLS is ideal for data analysis and enhancing model performance and robustness by focusing on the most relevant spectral regions. When compared to a reported HPLC method, the proposed methods showed non-significant difference regarding accuracy and precision. The developed models often yield faster results than HPLC. Once the model is built, it takes no time to predict multiple samples without requiring reconstruction, in addition, the proposed models minimize the costs of solvents and equipment compared to HPLC, making them a valuable option for quality control laboratories.

**Supplementary Information:**

The online version contains supplementary material available at 10.1186/s13065-025-01447-9.

## Introduction

**Fig. 1:**
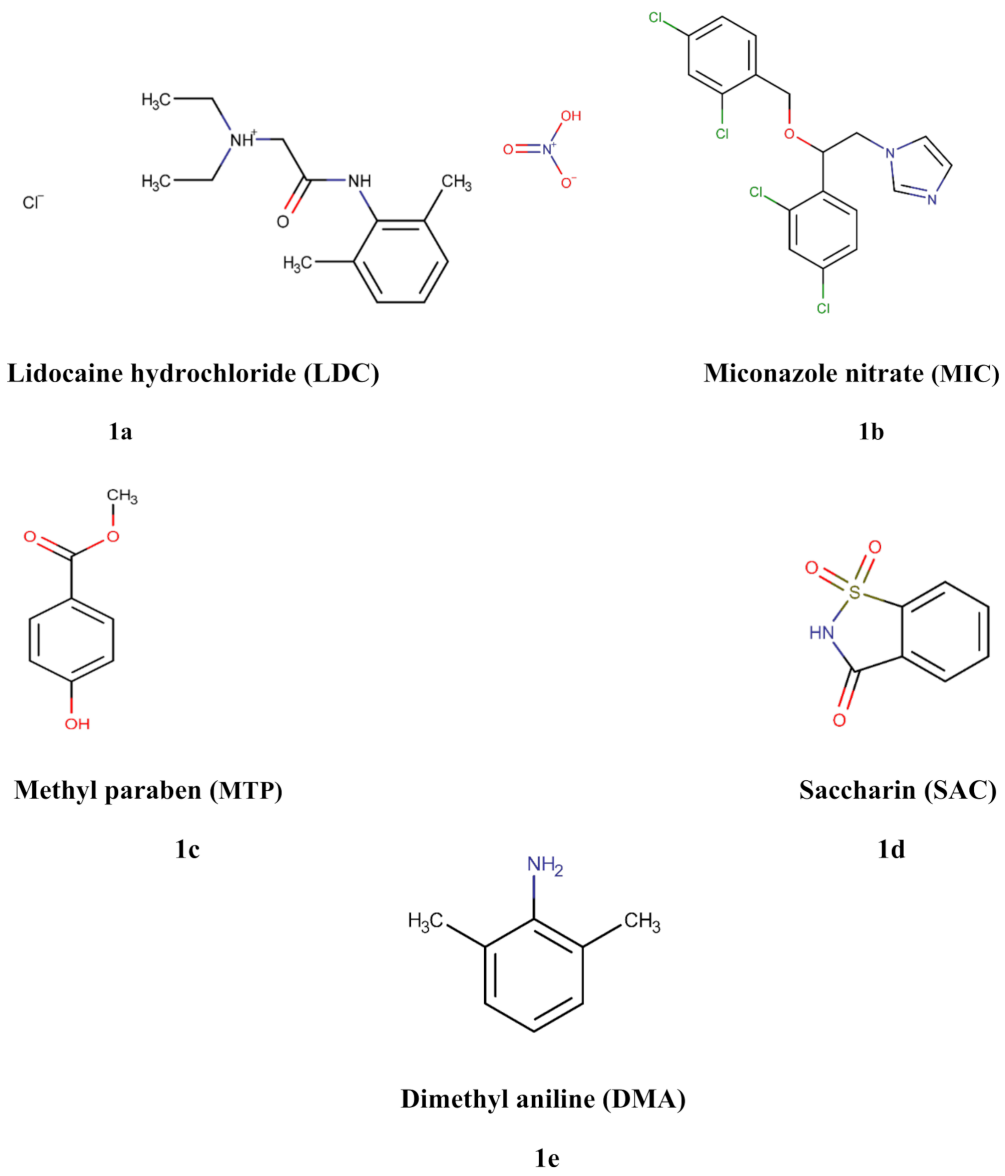
Chemical structures of (**a**) Lidocaine Hydrochloride (LDC). (**b**) Miconazole Nitrate (MIC). (**c**) Methyl Paraben (MTP). (**d**) Saccharin (SAC). (**e**) Dimethylaniline (DMA)

Lidocaine hydrochloride (LDC), chemically known as 2-(diethyl amino)-N-(2,6-dimethylphenyl) acetamide hydrochloride, Fig. 1a, is a topical local anesthetic applied to the mucous membranes and skin, numbing the area by blocking sodium ion currents, preventing nerve signals from being transmitted [1, 2]. Miconazole nitrate (MIC), chemically 1-[(2RS)-2-[(2,4-dichlorobenzyl) oxy]-2-(2,4-dichlorophenyl) ethyl)]-1 H-imidazole nitrate, Fig. 1b, is an imidazole fungicidal that works by inhibiting peroxidases, leading to peroxide accumulation and cell death, and by suppressing ergosterol formation [3]. LDC and MIC are combined as an antifungal oral gel, along with inactive ingredients; Methyl paraben (MTP) and saccharin sodium (SAC), it’s commonly used for the cure of gastrointestinal and oropharyngeal candidiasis [1].

Methyl paraben is methyl 4-hydroxybenzoate, Fig. 1c. It is widely used in various industries, as a preservative and antimicrobial agent [4]. Parabens, including MTP, have been linked with health issues like breast cancer and reproductive problems due to their endocrine-disrupting effects [5]. Some publications declare that these molecules are carcinogenic because they show estrogenic and antiandrogenic activity [5, 6]. Additionally, research has investigated the effects of the prenatal exposure of paraben on newborns’ birth weight and thyroid function. Consequently, parabens are now being classified as potentially harmful to health [7], guidelines specify that certain compounds should not be used, especially in items targeted for children, and if used, only in minimal amounts. Even so, the use of parabens as antimicrobial preservatives requires to be justified and monitored throughout the analysis and batch release, MTP is used in oral formulations at doses ranging from 0.015 to 0.2% [4].

Saccharin sodium, chemically known as 2 H-1λ6,2-benzothiazol-1,1,3-trione, Fig. 1d, is utilized in oral medicinal formulations as a non-nutritive sweetener. Given that, SAC is extracted together with the active components in the gel formulation, its separation is essential. Dimethyl aniline (DMA), lidocaine’s main impurity, also known as N, N-Dimethylaniline, Fig. 1e, is a pharmacologically inactive metabolite [1], it enters the body through skin mucosa and the mouth, potentially affecting organs such as the liver, heart, and kidneys [8]. Therefore, it is crucial to identify DMA in pharmaceutical dosage forms. According to the British Pharmacopoeia (BP), the acceptable limit for DMA is 0.01% [1].

A survey of the relevant literature indicated several methods that were used to analyze compounds under study either alone or in combination with other compounds. For LDC estimation, spectrophotometric [9], chromatographic [10, 11] and capillary electrophoretic [12] have been published, added to that, for the LDC with DMA, chromatographic [8], voltammetric [13], electrochemical [14] and spectrophotometric [15] methods have been reported. For MIC determination, voltammetry [16], spectrophotometry [17] and High-Performance Liquid Chromatography (HPLC) [18]. Besides, for MTP quantification, spectrophotometric [19], stability indicating- HPTLC [20] and electrochemical [21] methods have been published, in addition for quantitation of SAC, HPLC [22, 23]approaches have been reported. Based on our knowledge, there were three reported methods for determination of the two drugs LDC and MIC, which are HPLC–DAD for the quantification of the binary mixture in their pharmaceutical formulation [24]. HPLC and TLC for the determination of the mentioned mixture along with LDC potential impurity and an endocrine disruptor preservative [25].

As far as we know, there is no spectrophotometric method for resolution and determination of MIC and LDC along with dosage form preservatives; MTP and SAC, in addition to LDC potential impurity; DMA, because of their significantly overlapping spectra. Although spectrophotometry offers a wide range of advantages across various disciplines. It can identify minor changes in absorbance or light transmission, enabling accurate quantification of analytes at low concentrations. Also, it is non-destructive to samples and yields rapid results [26]. Integrating chemometrics with spectrophotometry is a forward step that enhances the analysis and interpretation of the overlapping spectra of the cited compounds [27, 28]. The developed models, especially biPLS effectively addressed the spectral overlap without requiring any prior separation, biPLS can efficiently identify the relevant spectral regions that aid in predicting the target variables while reducing the impact of noisy ones, leading to more reliable predictions with high accuracy and robustness. In addition, biPLS enhances model interpretability by focusing on significant intervals and excluding less informative ones [29].

Thus, our study aims to develop selective, accurate, and simple chemometric-assisted spectrophotometric methods (PCR, PLS, and biPLS) to simultaneously determine the two drugs along with the two preservatives in addition to the impurity in their laboratory prepared mixtures and gel formulation.

## Experimental

### Instruments and software

A Shimadzu 1650 UV-PC spectrophotometer (USA) with two identical 1.00 cm quartz cells was used. Scans were conducted in the range of 200.0–400.0 nm at 0.2 nm intervals, with a wavelength scanning speed of 2800 nm/min. The spectra were automatically generated by the Shimadzu UV-Probe 2.32 system software. Matlab^®^ version 7.0.1 (Mathworks Inc., 2004) and PLS_ Toolbox 2.1 were utilized for all data manipulations. The biPLS model was also constructed using the iToolbox.

## Materials

### Pure standards

Miconazole, lidocaine, saccharin and methyl paraben with certified purities of 100.37 ± 1.727, 100.16 ± 1.083, 98.00 ± 1.550 and 99.91 ± 0.855 respectively, were generously provided by Amriya Pharmaceutical Industries (Alexandria, Egypt). 2,6-Dimethylaniline, with a certified purity of 99.73 ± 0.838, was supplied by Sigma-Aldrich, St. Louis, MO.

### Pharmaceutical formulation

Micoban^®^ oral gel, batch no. 5,875,003, is claimed to contain 2.5% (w/w) MIC and 0.66% (w/w) LDC per gram of gel, in addition, the preservatives; MTP and SAC. It was produced by Amriya Pharmaceutical Industries in Alexandria, Egypt, and purchased from the local market.

### Chemicals and solvents

Methanol (Sigma Aldrich, Darmstadt, Germany).

#### Standard solutions

##### Stock standard solutions

Stock standard solutions of 1.0 mg/mL for LDC, MIC, SAC, MTP and DMA were prepared by accurately weighing 25.0 mg of each pure standard, transferring them to five separate 25-mL volumetric flasks, dissolving in methanol, and then filling to the mark with methanol [30].

#### Working standard solutions

Working standard solutions were prepared in methanol to obtain concentrations 100.00 µg/mL for LDC, MIC, SAC, MTP and DMA by transferring 10.0 mL from their corresponding stock standard solutions into 100-mL volumetric flasks and then filling to the mark with methanol.

### Procedures

#### Construction of calibration models

A five-factor, five-level experimental design with the concentration levels coded as -2, -1, 0, 1 & 2 was implemented [31]. 25 mixtures containing varying ratios of LDC, MIC, SAC, MTP and DMA were prepared. Eighteen mixtures were randomly chosen to construct the models (the calibration set), the mixtures were prepared by accurately transferring aliquots from the respective working standard solutions into 25-mL volumetric flasks and then filling to the mark with methanol. The produced mixtures were in the concentration ranges of (2.40–12.00 µg/mL) for LDC and MIC, (1.50–7.50 µg/mL) for DMA and MTP, and (2.00–6.00 µg/ mL) for SAC. The absorption spectra were scanned at intervals of 0.2 nm from 200.0 to 400.0 nm. The data was then exported and manipulated using Matlab^®^ with PLS Toolbox and iToolbox [32].

#### Validation of calibration models

The developed models were validated through the model development process and by external validation set after model development. During method development, cross validation was applied, leave one out, random subsets, contiguous blocks, and venetian blinds were tested, wherever the use of leave one out gave the best results. For external validation, a set of seven mixtures, each containing all five components, was selected at random. The concentrations of the prepared samples are shown in Additional file 1: Table S1.

#### Analysis of pharmaceutical dosage form

A 1.0 g sample of the gel formulation was accurately weighed into a 50-mL beaker and sonicated for 15 min with 30 mL methanol; it was then filtered into a 100-mL volumetric flask and the volume was brought up to the mark with the same solvent. From the prepared solution, aliquots were accurately transferred to 25-mL measuring flasks and filled to the mark with methanol to obtain a solution containing 12 µg/mL MIC and 3.2 µg/mL LDC. The absorption spectrum of this solution was recorded at 0.2 nm intervals in the range of 200.0–400.0 nm. The predicted concentrations were calculated using the developed models for the two drugs, the two preservatives and the impurity under study.

## Results and discussion

Due to the harmful effects of DMA on health and the ongoing debate about the impact of parabens on humans, animals, and ecosystems, it is increasingly important to separate and determine the hazardous DMA and MTP along with MIC and LDC.

**Fig. 2:**
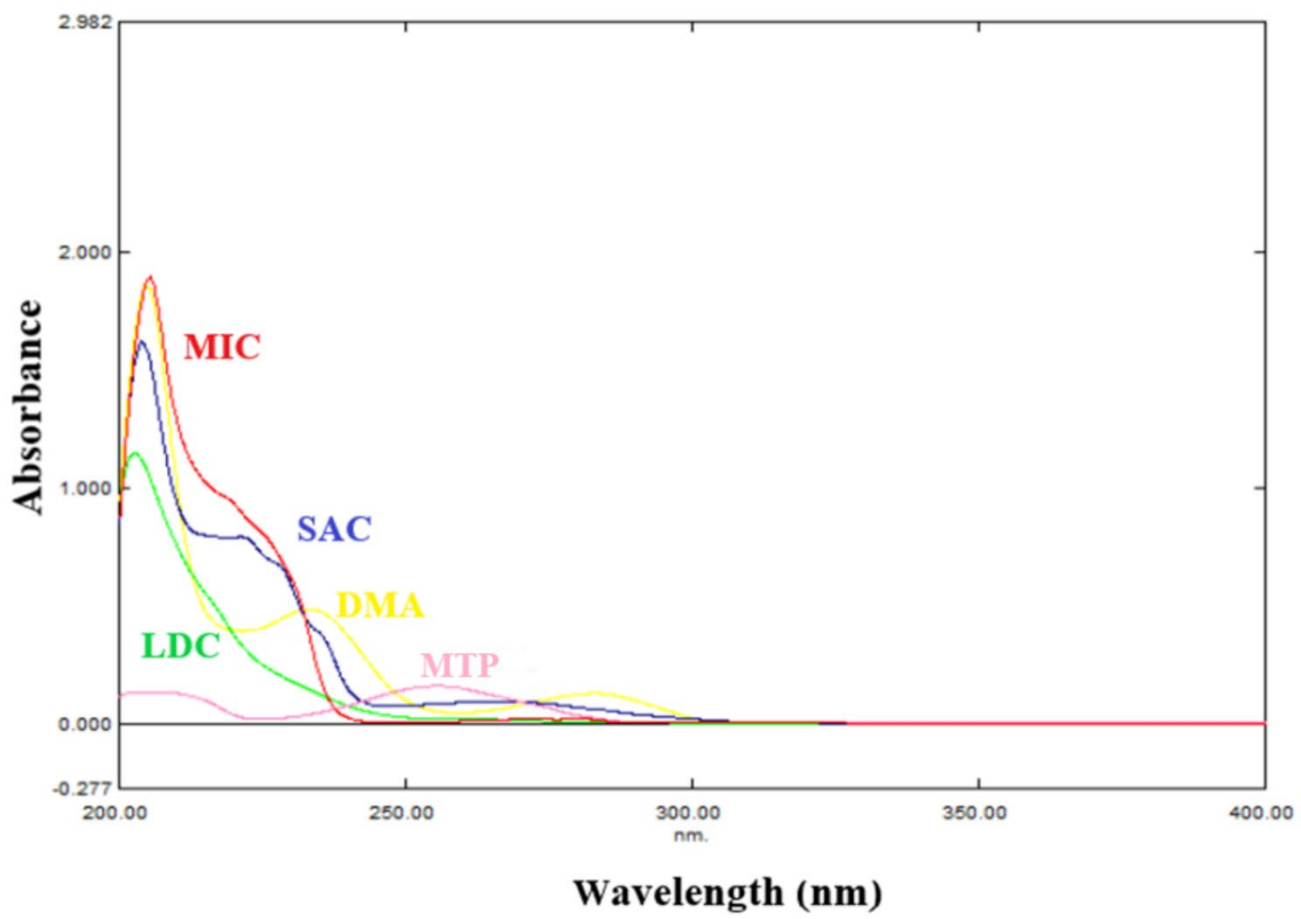
Absorption spectra of 12.00 µg/mL Miconazole Nitrate (MIC), 12.00 µg/mL Lidocaine hydrochloride (LDC), 8.00 µg/mL Methyl Paraben (MTP), 8.00 µg/mL Saccharin (SAC), and 8.00 µg/mL Dimethylaniline (DMA) using methanol as a solvent a

UV visible spectroscopy is a quick and simple method that has been applied extensively in numerous analytical applications, but univariate spectrophotometric techniques are unable to resolve the five components under investigation because of their severely overlapped spectra Fig. 2, which hinders their determination without the need for prior separation [33]. Chemometric-assisted spectrophotometric methods can help overcoming this by enabling the simultaneous resolution and determination of the two drugs, the preservatives, and the impurity despite of their overlapped spectra. The benefit of multicomponent analysis utilizing multivariate calibration is the rapidity of the process of determining the components of interest in a mixture since a separation step can be skipped [34].

Therefore, this study aims to create three different multivariate calibration models PLS, PCR, and biPLS. In quantitative pharmaceutical analysis, PCR and PLS models were commonly used to extract specific information from more general data [35]. biPLS as an advanced chemometric algorithm has shown superior precision and reliability when compared to the conventional PLS, so biPLS is introduced recently to all types of numerical data sets to enhance the models’ predictive performance [36].

A five factor, five-level experimental design was applied. The regression models were built using a training set (calibration set) of eighteen mixtures, the developed models were subjected to cross validation during their construction and an external validation set of seven samples after models’ development to test the prediction ability.

### Principal component regression (PCR) and partial least squares (PLS)

PCR and PLS are factor based full spectrum multivariate calibration models based on principal component analysis (PCA) which enable simple and rapid determination for complex samples, PLS uses an algorithm that analyses both absorbance and concentration matrices before capturing a number of latent variables (LVs), that encapsulate the bulk of raw data and use them to create a linear regression model [37, 38]. First, the raw data of the calibration mixtures were preprocessed using mean centering, then many cross-validation techniques were tried as random subsets, leave one out, contiguous blocks, and venetian blinds, the leave one out cross-validation approach yielded the best results, with the lowest RMSECV and the highest R-squared values. The lower the RMSECV and the higher the R-squared, the more closely a model can predict the actual observations. Instead of utilizing the full spectrum, a wavelength region is chosen, which can help with model simplification by removing nonlinear variables, yielding a correction model with fewer LVs and strong predictive power. The samples were scanned at 0.2 nm intervals with a spectral band of 220.0–280.0 nm, yielding 301 data points per each spectrum. The resulting spectral data matrix includes 25 rows for different samples and 301 columns for wavelengths (25 × 301).

Here, it is important to emphasize that the development of appropriate calibration models is required to lower the possibility of underfitting LVs, which occurs when not all relevant variance is accounted for. Conversely, incorporating too many LVs can lead to overfitting, as both systematic and noisy data are recorded, since LVs are calculated by the models using the F ratio. The optimal number of LVs that resulted in the lowest RMSECV during cross-validation were eight in both PCR and PLS models [39, 40]. The high number of LVs was correlated to the spectral similarity between LDC and its impurity; DMA which resulted in the occurrence of collinearity [41].

The residual error values were calculated for each component concentration were calculated and plotted against the true concentrations, Additional file 1: Figs. S1, S2, respectively. The applied regression models were assessed by the external validation set for prediction the five components, and the statistical and linear regression parameters are displayed in Table 1, indicating good prediction of the models, also satisfactory results were obtained by calculating the % recoveries for the components under study, Table 2.

**Table 1:** Statistical and linear regression parameters for the validation set using PCR and PLS models

Parameters	PCR					PLS				
	MIC	LDC	DMA	MTP	SAC	MIC	LDC	DMA	MTP	SAC
Mean recovery %	98.60	101.57	100.43	100.18	100.05	98.63	101.35	100.50	100.28	100.11
SD	1.779	2.083	1.342	0.922	2.288	1.759	1.132	1.128	0.761	1.943
RSD%	1.804	2.051	1.336	0.920	2.287	1.783	1.117	1.122	0.759	1.941
RMSEP	0.172	0.221	0.06	0.052	0.066	0.171	0.159	0.057	0.051	0.06
Correlation coefficient (r)	0.9980	0.9975	0.9970	0.9998	0.9988	0.9980	0.9991	0.9997	0.9998	0.9990
Slope	1.0133	0.9997	1.0058	1.0142	1.012	1.0159	1.0063	1.0043	1.013	1.0105
Intercept	-0.2061	0.1475	0.0028	-0.05	-0.036	-0.2239	0.0656	0.0101	-0.0425	-0.029
LOD^a^	0.440	0.596	0.177	0.124	0.223	0.477	0.348	0.168	0.126	0.203
LOQ^a^	1.450	1.805	0.536	0.377	0.676	1.444	1.055	0.510	0.382	0.615

^a^Calculated from equation [LOD (limit of detection) = 3.3 (SD/S), LOQ (limit of quantification) = 10 (SD/S); where SD is the standard deviation of regression residuals and S is the slope of the calibration curve

**Table 2:** Determination of the studied components in laboratory prepared mixtures in the validation set by the proposed PCR and PLS chemometric methods

Recovery %										
Mixture no.	PCR					PLS				
	MIC	LDC	DMA	MTP	SAC	MIC	LDC	DMA	MTP	SAC
1	98.89	103.89	101.11	100.67	98.75	98.89	102.92	101.11	100.67	99.00
8	100.69	99.17	99.33	100.00	99.00	100.56	100.21	99.33	100.00	99.00
9	98.96	99.33	100.33	99.67	100.60	98.96	99.67	100.33	99.67	100.60
12	97.08	100.42	101.78	100.67	102.20	97.08	101.35	101.78	100.67	102.00
14	97.64	101.08	101.50	101.60	101.50	97.64	102.08	101.33	101.60	100.50
15	100.83	104.06	100.93	98.67	96.00	101.00	101.15	100.93	99.33	97.00
20	96.11	103.02	98.00	100.00	102.33	96.25	102.08	98.67	100.00	102.67

### Backward interval partial least squares (biPLS)

The biPLS model is a characteristic variable selection method based on iPLS. The data set is split into equal-length intervals and then iPLS is applied, followed by backward elimination. This process involves removing one interval with the lowest RMSECV at a time, retaining the most relevant intervals that produce an optimal model in terms of RMSECV, finally the optimum combination with the lowest RMSECV was selected [29, 42].

The number of intervals is a crucial decision in biPLS. If there are too few intervals, the impacts of smaller peaks may be lost being that relatively broad spectral areas are analyzed each time. Conversely, too high intervals may result in excessive results on a local scale consuming a much longer computation time [43]. It will be possible to solve this problem by running biPLS multiple times with different interval numbers and deletion group compositions [29].

**Fig. 3:**
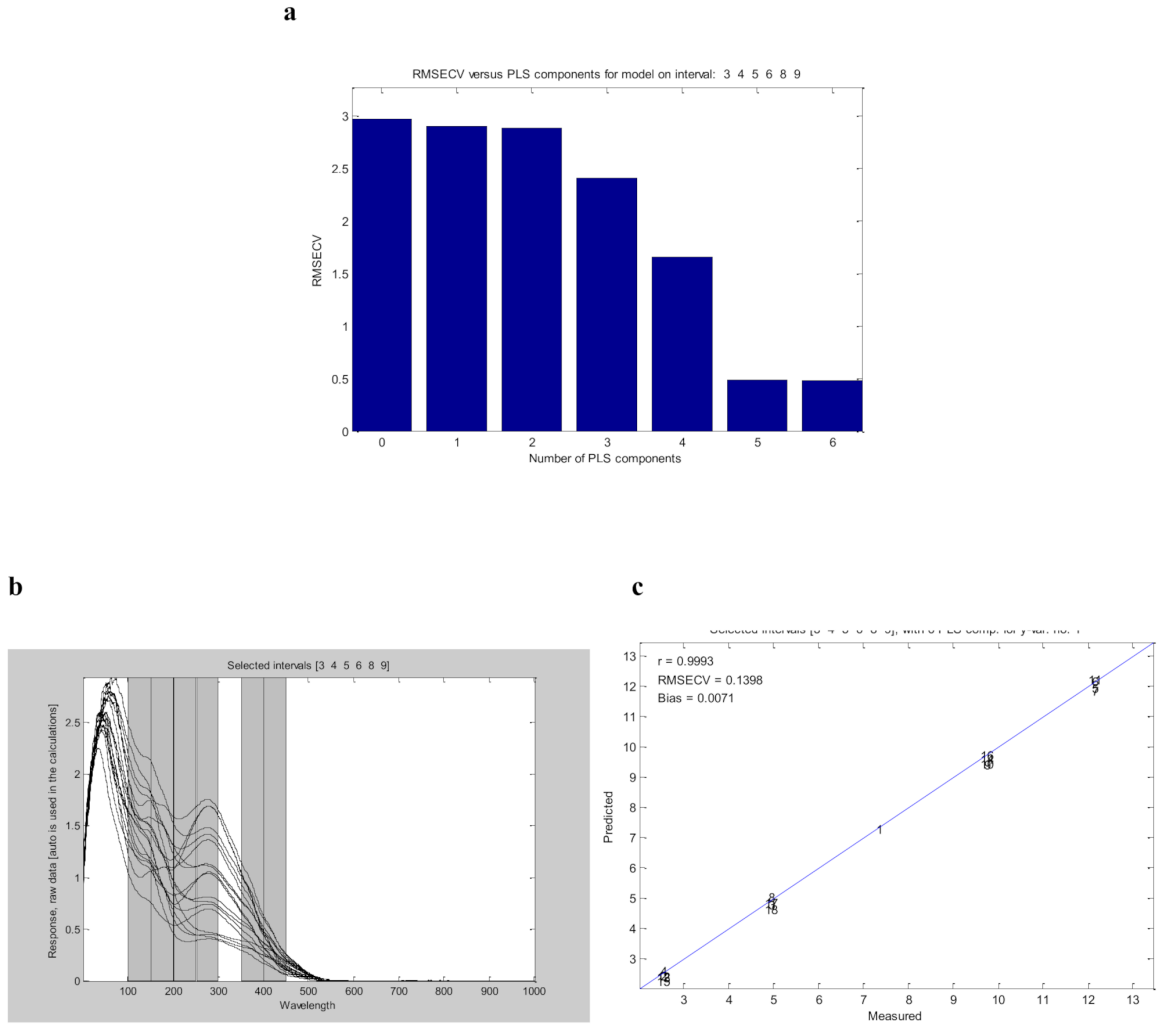
biPLS for intervals [3 4 5 6 8 9]: (**a**) RMSECV against PLS components; (**b**) biPLS model by combination of subintervals [3 4 5 6 8 9]; (**c**) true values of the five components (µg/mL) against the predicted values by cross‑validation for the biPLS model with 6 LVs

The biPLS model construction divides the entire spectral range (200.00–400.00 nm) into 20 equally distanced subintervals, each containing roughly 50 data points. A variety of combinations were selected and tested, numerous models are produced based on the number of intervals and the chosen combined intervals. The results are then automatically shown as the number and interval combinations of PLS components. Additionally, the RMSECV and r are calculated for the models. It was found that the combination of 6 subintervals [3, 4, 5, 6, 8, and 9] produced the best results with an acceptable number of LVs which is 6, RMSECV value of 0.1398 and *r* = 0.9993 as shown in Fig. 3. In comparison to PCR and PLS, the biPLS model is particularly effective in handling complex datasets with high collinearity and noise, it can resolve collinearity by determining the spectra’s most informative sections. Hence, biPLS is used for minimizing interference and improving the predictive performance of the models with a more effective determination using only six LVs instead of eight.

Spectrophotometry generally yields faster results because it involves simpler procedures and fewer steps than HPLC, which requires sample preparation, column equilibration, and longer run times. Moreover, it is typically more cost-effective as it doesn’t require expensive solvents, columns, or specialized equipment, and it also minimizes environmental impact by reducing the need for these chemical reagents and solvents [44, 45]. Particularly for chemometric models, once the model is built, it can quickly predict multiple samples without needing reconstruction. These advantages make chemometric-assisted spectrophotometric methods (such as PCR, PLS, and biPLS) an excellent choice for quality control laboratories [43, 46].

The concentrations of the five components were correlated with the residual error values, graphs are shown in Additional file 1: Fig S3. The linear and statistical regression parameters of the validation set are displayed in Table 3. Calculating the recoveries for the compounds under investigation yielded good results, Table 4.

**Table 3:** Statistical and linear regression parameters of the validation set using BiPLS model

Parameters	biPLS [3 4 5 6 8 9]				
	MIC	LDC	DMA	MTP	SAC
Mean recovery %	99.21	100.83	100.14	99.89	100.34
SD	1.497	2.252	1.476	1.158	1.775
RSD%	1.509	2.233	1.474	1.159	1.769
RMSEP	0.131	0.196	0.053	0.052	0.052
Correlation coefficient (r)	0.9986	0.9972	0.9996	0.9998	0.9997
Slope	1.0217	1.0093	0.9945	1.0161	1.029
Intercept	-0.2226	-0.0027	0.06	-0.07	-0.082
LOD^a^	0.390	0.700	0.180	0.140	0.100
LOQ^a^	1.180	2.110	0.540	0.420	0.300

^a^Calculated from equation [LOD (limit of detection) = 3.3 (SD/S), LOQ (limit of quantification) = 10(SD/S); where SD is the standard deviation of regression residuals and S is the slope of the calibration curves

**Table 4:** Determination of the studied components in laboratory prepared mixtures in the validation set by the proposed BiPLS chemometric method

Recovery %					
Mixture no.	biPLS				
	MIC	LDC	DMA	MTP	SAC
1	98.06	100.42	100.89	100.44	101.25
8	100.83	99.58	99.20	99.33	100.60
9	99.38	100.00	100.00	99.17	101.40
12	98.23	99.17	101.56	100.80	101.60
14	99.44	100.50	101.33	101.47	101.00
15	101.33	107.08*	100.67	98.00	96.50
20	97.22	105.31	97.33	100.00	100.00

* Rejected value according to Q rejection rule [42]

**Fig. 4:**
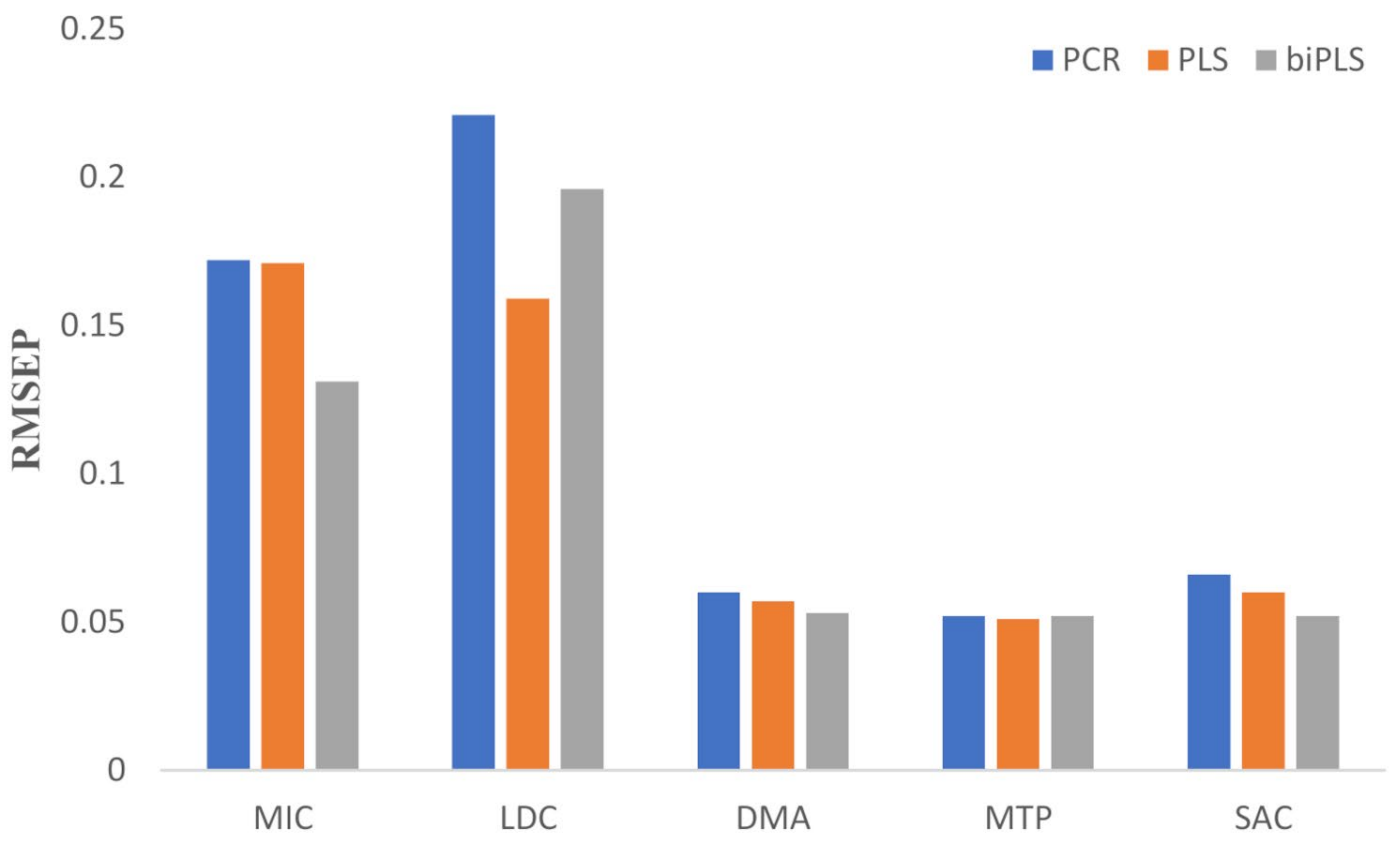
Comparison of RMSEP of the five components between the three proposed chemometric methods

Figure 4 compares the RMSEP values for the three models of the five components under study, demonstrating the superiority of biPLS over PCR and PLS models. biPLS showed the lowest RMSEP values, making it the most efficient model with the fewest latent variables.

### Application to pharmaceutical dosage form

The proposed multivariate chemometric models were used for the determination of MIC and LDC in their combined dosage form; Micoban oral gel^®^. Samples were scanned and the data were processed as specified by each model. The recovery and standard deviation values are shown in Table 5. The models were successfully applied for predicting the concentration of the studied compounds. Furthermore, the methods determined the two preservatives, MTP and SAC, which were found to be 0.18% and 0.23% respectively. DMA, the impurity, was not detected in the dosage form.

**Table 5:** Determination of MIC and LDC in Micoban oral gel^®^ using the proposed chemometric methods

Pharmaceutical formulation	Compound	PCR	PLS	biPLS
		Recovery^a^% ± SD		
Micoban oral gel^®^	MIC	99.23 ± 0.191	99.08 ± 0.177	97.56 ± 0.332
	LDC	97.67 ± 0.643	96.51 ± 0.948	99.14 ± 1.174

^a^Mean of three determinations

### Statistical comparison

The results obtained using the proposed models for determination the two drugs in Micoban oral gel^®^ were statistically compared to those obtained by applying a reported HPLC method [24]. There was no significant difference in both accuracy and precision between the proposed methods and the reported one [47], as indicated by the calculated values of the t and F tests being lower than their respective theoretical values as shown in Table 6.

**Table 6:** Statistical comparison of the results obtained by the proposed methods and a reported method for the determination of MIC and LDC in their pharmaceutical formulation

Value	PCR		PLS		BiPLS		Reported method^a^[24]	
	MIC	LDC	MIC	LDC	MIC	LDC	MIC	LDC
Mean	99.23	97.67	99.08	96.51	97.56	99.14	98.42	98.27
SD	0.191	0.643	0.177	0.948	0.332	1.174	0.641	1.684
*n*	3	3	3	3	3	3	3	3
Variance	0.0364	0.4134	0.0313	0.8987	0.1102	1.3782	0.4109	2.8359
Student’s t-test^b^ (2.776)	2.098	0.577	1.719	1.577	2.063	0.734		
F value^b^ (19.00)	11.29	6.86	13.13	3.16	3.73	2.06		

^a^Gradient HPLC-DAD stability indicating determination of miconazole nitrate and lidocaine hydrochloride in their combined oral gel dosage form

^b^The tabulated values of t and F at *P* = 0.05 are represented by figures between parentheses

## Conclusion

Overall, this work has proven the value of multivariate calibration in overcoming spectral overlapping challenges. The proposed models are highly reproducible, accurate, time saving and cost effective. Thus, they are simply applicable to the quick routine analysis of the compounds under study either in their pure form and in dosage form, in quality control laboratories. The methods’ validity has also been confirmed by the successful results obtained. The biPLS model outperformed the PCR and PLS models in determining co-formulated LDC, MIC, SAC, and MTP, as well as DMA, without prior separation, due to its lower latent variables and prediction error.

## Electronic supplementary material

Below is the link to the electronic supplementary material.

Supplementary Material 1

## Data Availability

All data analyzed during this study are included in this published article and raw data are available from the corresponding author upon reasonable request.

## Abbreviations

MIC: Miconazole nitrate
LDC: Lidocaine hydrochloride
MTP: Methyl paraben
SAC: Saccharin sodium
DMA: Dimethyl aniline
BP: British pharmacopoeia
PCR: Principal component regression
PLS: Partial least squares
biPLS: Backward interval partial least squares
