# Supplementary material for: Chemometric-assisted UV spectrophotometric methods for determination of miconazole nitrate and lidocaine hydrochloride along with potential impurity and dosage from preservatives
Source: BMC Chem. 2025 Mar 28;19(1):82. doi: 10.1186/s13065-025-01447-9 (PMC11954262; doi:10.1186/s13065-025-01447-9)
Supplement: Supplementary file 1 — Supplementary Material 1 [file 13065_2025_1447_MOESM1_ESM.docx]

**Additional Information for**

**Chemometric-assisted UV spectrophotometric methods for determination of miconazole nitrate and lidocaine hydrochloride along with potential impurity and dosage from preservatives**

**Table S1: Concentrations in µg/mL of MIC, LDC, DMA, MTP, and SAC in the calibration and validation sets**

| **Samples** | **Concentration (µg/mL)** | | | | |
| --- | --- | --- | --- | --- | --- |
|  | **MIC** | **LDC** | **DMA** | **MTP** | **SAC** |
| 1 | 7.20 | 7.20 | 4.50 | 4.50 | 4.00 |
| 2 | 7.20 | 2.40 | 3.00 | 1.50 | 6.00 |
| 3 | 2.40 | 4.80 | 1.50 | 7.50 | 6.00 |
| 4 | 4.80 | 2.40 | 7.50 | 7.50 | 4.00 |
| 5 | 2.40 | 12.00 | 7.50 | 4.50 | 3.00 |
| 6 | 12.00 | 12.00 | 4.50 | 3.00 | 6.00 |
| 7 | 12.00 | 7.20 | 3.00 | 7.50 | 3.00 |
| 8 | 7.20 | 4.80 | 7.50 | 3.00 | 5.00 |
| 9 | 4.80 | 12.00 | 3.00 | 6.00 | 5.00 |
| 10 | 12.00 | 4.80 | 6.00 | 6.00 | 4.00 |
| 11 | 4.80 | 9.60 | 6.00 | 4.50 | 6.00 |
| 12 | 9.60 | 9.60 | 4.50 | 7.50 | 5.00 |
| 13 | 9.60 | 7.20 | 7.50 | 6.00 | 6.00 |
| 14 | 7.20 | 12.00 | 6.00 | 7.50 | 2.00 |
| 15 | 12.00 | 9.60 | 7.50 | 1.50 | 2.00 |
| 16 | 9.60 | 12.00 | 1.50 | 1.50 | 4.00 |
| 17 | 12.00 | 2.40 | 1.50 | 4.50 | 5.00 |
| 18 | 2.40 | 2.40 | 4.50 | 6.00 | 2.00 |
| 19 | 2.40 | 7.20 | 6.00 | 1.50 | 5.00 |
| 20 | 7.20 | 9.60 | 1.50 | 6.00 | 3.00 |
| 21 | 9.60 | 2.40 | 6.00 | 3.00 | 3.00 |
| 22 | 2.40 | 9.60 | 3.00 | 3.00 | 4.00 |
| 23 | 9.60 | 4.80 | 3.00 | 4.50 | 2.00 |
| 24 | 4.80 | 4.80 | 4.50 | 1.50 | 3.00 |
| 25 | 4.80 | 7.20 | 1.50 | 3.00 | 2.00 |

*Samples 1, 8, 9, 12, 14, 15, and 20 are those selected for external validation

|  |  |
| --- | --- |
|  |  |
|  | |

**Fig. S1: Residual error values versus the true concentration for the studied components using PCR method [X- axis is the true concentration (µg/mL), Y-axis is the error = (true concentration – found concentration)]**

|  |  |
| --- | --- |
|  |  |
|  | |

**Fig. S2: Residual error values versus the true concentration for the studied components using PLS method [X- axis is the true concentration (µg/mL), Y-axis is the error = (true concentration – found concentration)]**

|  |  |
| --- | --- |
|  |  |
|  | |

**Fig.S3: Residual error values versus the true concentration for the studied components using biPLS method [X- axis is the true concentration (µg/mL), Y-axis is the error = (true concentration – found concentration)]**
